# Mining a Crowdsourced Dictionary to Understand Consistency and Preference in Word Meanings

**DOI:** 10.3389/fpsyg.2019.00268

**Published:** 2019-02-18

**Authors:** Brendan T. Johns

**Affiliations:** Department of Communicative Disorders and Sciences, University at Buffalo, Buffalo, NY, United States

**Keywords:** distributional semantics, semantic memory, big data, corpus studies, knowledge acquisition

## Abstract

Big data approaches to psychology have become increasing popular (Jones, 2017). Two of the main developments of this line of research is the advent of distributional models of semantics (e.g., Landauer and Dumais, 1997), which learn the meaning of words from large text corpora, and the collection of mega datasets of human behavior (e.g., The English lexicon project; Balota et al., 2007). The current article combines these two approaches, with the goal being to understand the consistency and preference that people have for word meanings. This was accomplished by mining a large amount of data from an online, crowdsourced dictionary and analyzing this data with a distributional model. Overall, it was found that even for words that are not an active part of the language environment, there is a large amount of consistency in the word meanings that different people have. Additionally, it was demonstrated that users of a language have strong preferences for word meanings, such that definitions to words that do not conform to people’s conceptions are rejected by a community of language users. The results of this article provides insights into the cultural evolution of word meanings, and sheds light on alternative methodologies that can be used to understand lexical behavior.

## Introduction

An emerging area within the psychological and cognitive sciences is the use of big data to develop and analyze theories of cognition (Jones, 2017; Johns et al., in press). One of the key developments in big data approaches to cognition is the emergence of distributional models of semantics, which learn the meaning of words from statistical patterns contained in very large sources of texts (see Jones et al., 2015 for a review). The original, and best known, model of this class is Latent Semantic Analysis (LSA; Landauer and Dumais, 1997), which spurred the development of many new approaches (e.g., Lund and Burgess, 1996; Griffiths et al., 2007; Jones and Mewhort, 2007; Shaoul and Westbury, 2010; Mikolov et al., 2013; Jamieson et al., 2018). The insight that these models exploit is that lexical semantic behavior seems to be systematically related to the co-occurrence of words within the natural language environment.

The representations derived from distributional models have been used to examine behavior across a number of domains, including lexical organization (Jones et al., 2012; Hoffman et al., 2013; Hsiao and Nation, 2018; for a review, see Jones et al., 2017), episodic memory (Johns et al., 2012b, 2014; Mewhort et al., 2017), morphological processing (Marelli and Baroni, 2015; Marelli et al., 2017; Westbury and Hollis, 2018), lexical-perceptual integration (Andrews et al., 2009; Johns and Jones, 2012; Lazaridou et al., 2017), prediction (Frank and Willems, 2017), decision (Bhatia and Stewart, 2018), and sentence processing (Johns and Jones, 2015). Furthermore, they have begun to be used not just as theories of human behavior, but as analysis tools to quantify linguistic data. For example, Johns and Jamieson (2018) analyzed a large sample of fiction books to understand individual variance in language usage. Taking a different applied approach, Johns et al. (2018b) used a distributional model to analyze the changes that were occurring in patients who were developing a cognitive impairment, while Taler et al. (2019) used a distributional model to assess differences in memory retrieval performance across tens of thousands of participants spanning the aging spectrum. Green et al. (2014, 2015) used LSA as a method to understand the history of psychology, through the analysis of abstracts from early issues of *Psychological Review*. Additionally, distributional models have been key in the development of automated essay marking technology (see Jones and Dye, 2018, for a review). Finally, these models have been particularly impactful in computational linguistics, where they have been used to shed important light on quantitative aspects of word meanings (see Levy and Goldberg, 2014; Levy et al., 2015, for important examples).

In terms of theory development, big data approaches to cognition are largely abductive in nature (see Haig, 2005, in press, for a general discussion of the use of abduction in psychological science and Johns et al., in press, for a specific discussion of abduction to theoretical developments in cognition). In abduction, hypotheses are generated to “found” data. That is, theories are not used to inform data collection, but instead theories are formed in response to the patterns seen in data, using within-domain knowledge. An example of abduction in psychological science is provided by modern theories of lexical organization. Specifically, in response to a large collection of lexical decision and naming being publically released [e.g., the English lexicon project (ELP); Balota et al., 2007], a number of new models have been developed to explain the patterns in this data at the item-level (e.g., Adelman et al., 2006; Johns et al., 2012a; Jones et al., 2012; Hollis, 2017; for a review, see Jones et al., 2017).

An example model in the area of lexical organization that used the abductive approach to spur new empirical research is the Semantic Distinctiveness Model of Jones et al. (2012), which weights word occurrences based on how semantically unique the usage of a word is (compared to a word frequency count, where each occurrence of a word is weighted equally). It was found that this model provided a better fit to the lexical decision time data from the ELP better than alternative models. This model was then used to generate hypothesis, which were then be subjected to targeted experimentation (e.g., Johns et al., 2016a,b; see Hsiao and Nation, 2018 for a similar approach). That is, a new model was abducted from a mega dataset of human behavior, which was then tested and modified using standard experimental methodologies (see the section “Discussion” for a further discussion of this issue).

The goal of the current article is to further this approach by applying a distributional analysis to mined data from an online, crow-sourced dictionary, namely the Urban Dictionary, in order to gain an understanding of the consistency and preference that people have for word meanings. Urban Dictionary (UD) is a website where users of the website generate definitions to slang words and phrases, or alternative definitions to known words. Users also generate examples usages of the defined word. Additionally, other UD users can vote on how accurate they believe a definition is, by giving the definition either a thumbs up or thumbs down. This article will serve as a prime example of abduction in psychological science, as the analysis described below will detail the use of semantic memory “in the wild.” Specifically, the definitions that users of the UD generate will be analyzed to determine if there are systematic tendencies in the meanings that people have acquired to different words, with the hope that these patterns can spur empirical and theoretical developments in the study of lexical semantics.

The use of data mined from online websites to gain a better understanding of psychological constructs is not without precedence. For example, Schwartz et al. (2013) used Facebook messages to assess how language usage differs by the personality of an individual. Similarly, Park et al. (2016) used Facebook messages to demonstrate gender differences in the usage of language. Herdağdelen and Marelli (2017) used Facebook and Twitter posts to build lexical norms. Thus, the use of UD data fits well with current trends in the psychological and cognitive sciences.

The task set to users of UD is somewhat similar to a classic experimental task in cognitive psychology, namely free association. In a standard free association task, a subject is asked to generate as many related words as they can to a cue word or concept (Nelson et al., 2000). The resulting data provides insight into the underlying semantic representation that a person has about a word or concept, through feature overlap to other words or concepts. A UD user who is generating a definition to a word is doing something similar, albeit more directly, by providing a sentence that states what they believe a word (i.e., the cue) means and how it is used. Mining UD data provides an opportunity to analyze this data type, and the representations contained in semantic memory more generally, at scale.

Additionally, this type of data provides an opportunity to study the evolution of word meaning at a stage not previously done before. An emerging viewpoint within the language sciences is that language is a complex adaptive system which serves a fundamentally social and communicative purpose (Christiansen and Chater, 2008; Beckner et al., 2009). A language that is adaptive entails that different language users should communicate in similar ways as other users of that language, given common social and cultural backgrounds. The data contained in the UD provides a look into language formation at an early stage, by allowing users to generate definitions to words and phrases that are not all currently an active part of the language environment, at least to most users of the English language. That is, it provides an insight into the processes by which meaning to new words are developed and communicated within and across groups.

For example, the word *jocking* has no entries in a standard dictionary, but has 24 entries in the UD. Some UD users propose that it means to engage in flirtatious behavior with another. This is not the only definition, however; other users propose that it instead means to copy another’s personal style. An alternative definition entails that it means to place yourself above your current social status. Others have more unsavory definitions in mind. That is, there seems to be active competition within UD definitions: different users are attempting to communicate the meaning of a word that is unknown to most, and many have different ideas about what a specific word or phrase means. The definitions that other users feel is the most representative of its meaning receive the most positive ratings (i.e., thumbs up), while definitions that do not cohere to other user’s expectations are given negative ratings (i.e., thumbs down). Thus, there are social and communicative pressures placed on how receptive UD users are to a proposed definition.

By analyzing the definitions that are generated by different users, it provides insight into the consistency of semantic representations across a large sample of people using a naturalistic stimuli and task at the beginnings of language formation. By examining how well received these definitions are by the general community, it allows for a determination of how receptive the community is to a user’s conception of what a word means. Taken together, the data contained in the UD provide an opportunity to study the consistency and preference that people have for word meanings. This will be accomplished by using a distributional model to analyze a large amount of mined data from the UD, as detailed below.

## Materials and Methods

### Mining UD Data

To mine UD data, the Urban Dictionary API^1^ was used to extract five fields: (1) the word being defined (i.e., the cue word), (2) definition of the cue word, (3) example usage of the cue word, (4) number of thumbs up to the definition, and (5) number of thumbs down to the definition. In total, 3 million definitions were mined. To simplify the analysis and allow for a comparison of the consistency of the definitions produced (the main goal of this analysis), only cue words that had at least two different definitions were included in the analysis, in order for an analysis of the intra-similarity of definitions to be possible. In order to avoid a cue word from dominating resulting similarity distributions, the number of possible definitions for a single cue word was cut off at 150. Additionally, definitions and examples had to contain at least 2 content words (i.e., non-function words; defined as being words that are not in the stop list used by Landauer and Dumais, 1997). The actual word (or words) being defined were also removed from any definition or example, to ensure that similarity across definitions/examples is not simply due to the overlapping presence of the cue word(s).

Additionally, to be included in the analysis, a definition had to have at least five cumulative thumb up or thumb down ratings. This cutoff simply ensures that all cue words being analyzed was of interest to some other users.

Since not all cue words had examples given for them, there are more definitions than examples contained in this analysis. For definitions, there were 106,603 different cue words with 434,011 definitions. For example usages, there were 90,685 different cue words with 385,986 examples. Cues that consisted of more than one word were included in the general analysis (e.g., the multi-word cue *couch potato* is contained in the dataset).

To determine how established the cue words from the UD data are, the number of cue words that were contained in the English lexicon project (Balota et al., 2007), a standard list of words contained in English, was calculated. The English lexicon project contains 40,481 words in total. It was found that of the 106,603 cue words, only 11,083 were contained in the English lexicon project (i.e., approximately 10% of the cue words being defined were likely of common parlance). This suggests that many of the words that are contained in the UD data are newly emerging words, which may have considerable variability in terms of the meaning that different individuals have acquired for those words. In the simulations contained below, this split between words from the ELP and words not contained in the ELP will be used to contrast established words from the likely newly emerging words contained in the UD data.

Descriptive statistics of the definitions and examples attained from the UD is contained in Table 1. This table includes the number of words, number of content words, number of unique words, and the number of unique words that were contained in the English lexicon project (Balota et al., 2007). By assessing the number of words that were contained in the ELP, it provides insight into how many of the words used are relatively common words in the English language. This table shows that definitions are generally longer than examples. Additionally, the majority of the unique words contained in both the definitions and examples were words from the ELP, suggesting that the words UD users use for their definitions and examples come from mainstream English.

**Table 1:** Descriptive statistics of UD definition and examples.

	# of words	# of content words	# of unique content words	# of ELP content words
Definitions	30.95	15.06	13.667	12.258
Examples	18.25	10.13	9.1	7.99


As an example of the type of information that is contained in the mined data, Table 2 contains four entries for the word *dance*. The entries in the table show that there is consistency across both the definitions and examples generated to this cue word. Of course, *dance* is a well know word compared to most cue words in the UD, so that consistency may not hold for every cue, as the previous discussion of the word *jocking* shows.

**Table 2:** Example UD entries for the word dance.

Definition	Example usage	+	-
An art form of expression using movement.	Look at that girl dance!	378	104
The manipulation of movement.	Would you care to dance?	725	258
A well-known symptom or side effect of listening to music.	I laughed so hard when Tam danced the other night.	66	42
A form of activity, with many different styles. Dance can be taught at studios or non-formally as well. It’s a good expression of emotion and feelings.	I’m going to my dance classes tonight.	620	300


Given that the language contained in the UD data is likely significantly different than other language sources, a training word list for a distributional model was constructed by determining the 150,000 most frequent words across the cue words, definitions, and example usages. Using this list allows for most of the words in the UD definitions and examples to be accounted for.

### Distributional Model

The distributional model used in this analysis will be the BEAGLE model of semantics (Jones and Mewhort, 2007). Broadly, it works by “reading” a text corpus and, en route, encoding each word’s meaning into a set of corresponding vectors. The theory is one in a larger class of distributional semantic models (Landauer and Dumais, 1997; Griffiths et al., 2007; Mikolov et al., 2013). BEAGLE processes language at the sentence level.

Mechanistically, BEAGLE is expressed in algebra. At the outset of a simulation, each of the unique words in the model’s vocabulary is represented by a unique *n*-dimensional environment vector, *e*, with each element assigned a random deviate from a normal distribution with mean zero and variance $\sqrt{1/n}$ (in the simulations that follow, dimensionality was set to *n* = 2,048). Environment vectors are stable over a simulation and are meant to serve as unique identifiers for the words in the corpus. These environment vectors are used to build up a memory representation for a word, *m*.

The memory vector for each word is composed of two kinds of information: context information and order information. A context vector, *c*, is computed by summing the environmental vectors for all other words in the same sentence (i.e., excluding the word of interest) into the representation for that word:

(1) $$c_{i}=\sum_{j=1}^{n}e_{j}$$

where *c* is the context vector formed for word *i* in the sentence, and *j* goes through each of the other words contained in the sentence. The context vector is then used to update a word’s representation in memory:

(2) $$m_{i}=m_{i}+c_{i}$$

Summing the environment vectors in this manner causes the memory vectors for all words in the same sentence to grow more similar to one another.

Order information is used to learn how a word is used within a sentence and is computed by encoding the *n*-grams (up to a certain size) that surround a word within a sentence. The computation of order information relies on non-commutative circular convolution (Plate, 1995). A convenient property of circular convolution is that it constructs a unique vector from the combination of two input vectors; this unique vector represents the association between those vectors.

Circular convolution is used to construct unique *n*-gram representations of words in sentences. To learn this information, BEAGLE applies circular convolution recursively to words in a sentence:

(3) $$o_{i}=\sum_{j=1}^{\mathrm{p\lambda}-(p^{2}-p)-1}bind_{\mathrm{ij}}$$

where, *o*_i_ is the order information for word *i, p* is the position of word *i* in the sentence, λ is the breadth of the binding (e.g., if λ = 3, the model encodes bigrams and trigrams but not higher order units), and *bind*_ij_ is the convolution between word *i* in a sentence and word *j* in the sentence. Consistent with Jones and Mewhort (2007), the λ parameter was set at 7 in this analysis.

To illustrate the operation, the order information for the word dog, *o*_dog_, in the sentence, “a dog bit the mailman,” is encoded as a sum of the following (example taken from Jones and Mewhort, 2007),

$$\begin{array}{cc} \begin{array}{l} bind_{\mathrm{dog},\;1}=e_{a}⊛\Phi \\ bind_{\mathrm{dog},\;2}=\Phi ⊛e_{\mathrm{bit}} \end{array}\} & Bigrams \end{array}$$

$$\begin{array}{cc} \begin{array}{l} bind_{\mathrm{dog},\;3}=e_{a}⊛\Phi ⊛e_{\mathrm{bit}}\\ bind_{\mathrm{dog},\;4}=\Phi ⊛e_{\mathrm{bit}}⊛e_{\mathrm{the}} \end{array}\} & Trigrams \end{array}$$

$$\begin{array}{cc} \begin{array}{l} bind_{\mathrm{dog},\;5}=e_{a}⊛\Phi ⊛e_{\mathrm{bit}}⊛e_{\mathrm{the}}\\ bind_{\mathrm{dog},\;6}=\Phi ⊛e_{\mathrm{bit}}⊛e_{\mathrm{the}}⊛e_{\mathrm{mailman}} \end{array}\} & Quadgrams \end{array}$$

$$\begin{array}{cc} bind_{\mathrm{dog},\;7}=e_{a}⊛\Phi ⊛e_{\mathrm{bit}}⊛e_{\mathrm{the}}⊛e_{\mathrm{mailman}}\} & Tetragram \end{array}$$

where ⊛ denotes circular convolution and Φ is a universal placeholder used in the computation of order information for every word in every position in every sentence (i.e., Φ is a random environment vector constructed in the same way as the other environment vectors). Once computed, the order vector is summed into the word’s central representation (i.e., *m*_dog_ = *m*_dog_ + *o*_dog_), equivalent to what is done for context information in Equation (2). In this article, the representation used will be the composite of the order and context vectors.

At the end of training, BEAGLE has learned both paradigmatic and syntagmatic information about a word’s usage. It has been shown to account for a variety of lexical semantic behaviors (e.g., Jones and Mewhort, 2007; Johns et al., 2016a, 2018b; Recchia et al., 2015; Johns and Jamieson, 2018).

Most uses of distributional models rely upon comparing the meaning of one word to another word, for instance to account for word similarity data. However, in this study the goal is to compare the meaning of a sentence (or multiple sentences) to one another. To form the meaning of a definition or example from the UD data, a discourse vector, *d*, will be constructed by summing the memory vectors of all the words in a definition or example:

(4) $$d_{i}=\sum_{j=1}^{n}m_{j}$$

As stated previously, only content words will be summed, defined as words that are not on the stop list of Landauer and Dumais (1997). The discourse vector will represent the overall meaning of a definition or example. Johns and Jamieson (2018) recently used a similar method to compare the similarity of even larger units of language, namely whole books.

To compare the semantic content of two discourses, a similarity value will be attained by taking the vector cosine (normalized dot product) between two discourse vectors. A vector cosine provides a value between -1 and 1, with 1 meaning that the vectors are perfectly overlapping, and -1 signaling that two vectors contains values that are in exact opposition. Discourse similarity will be the primary data used in the below analyses.

### Training Corpus

The corpus used to train the BEAGLE model was composed of writings from a number of different sources, with the hope that having a diverse training corpus would allow for many of the words used in the UD data to be included. The corpus included Wikipedia articles (Shaoul and Westbury, 2010), Amazon product descriptions (attained from McAuley and Leskovec, 2013), television and movie subtitles, fiction books, non-fiction books, and young adult books. See Johns et al. (2018a) for a more detailed analysis of these language sources. In total, the corpus consisted of approximately 160 million sentences with 1.4 billion words. Additionally, of the 150,000 most frequent words contained in the UD data, approximately 145,000 of them were also used in the corpus. This means that the vast majority of the words contained in the UD data had semantic representations derived for them.

### Analysis Technique

The main metric assessed in this analysis will be the similarity between the definitions and example usages that different users constructed to the same cue, as a way of determining how similar the meaning that two people have about the same word are. The similarity values taken between definitions/examples of the same cues will be used to form distributions of (typically) millions of comparisons. These will be referred to as intra-word similarity distributions.

However, in order to get a sense of how similar the definitions produced to the same word are, it is necessary to have a comparison distribution. A comparison distribution will be constructed by randomly sampling two definitions or examples generated to different cue words and taking the similarity between them. The resulting distribution will be referred to as extra-word similarity distributions. This distribution is an attempt to quantify the random similarity of UD definitions and examples. Any positive shift over this distribution would signal that the language being compared in that distribution exceeds the similarity of randomly selected definitions or examples. There will be one million random samples contained in the extra-word comparison distribution for each comparison.

## Results

**FIGURE 1:**
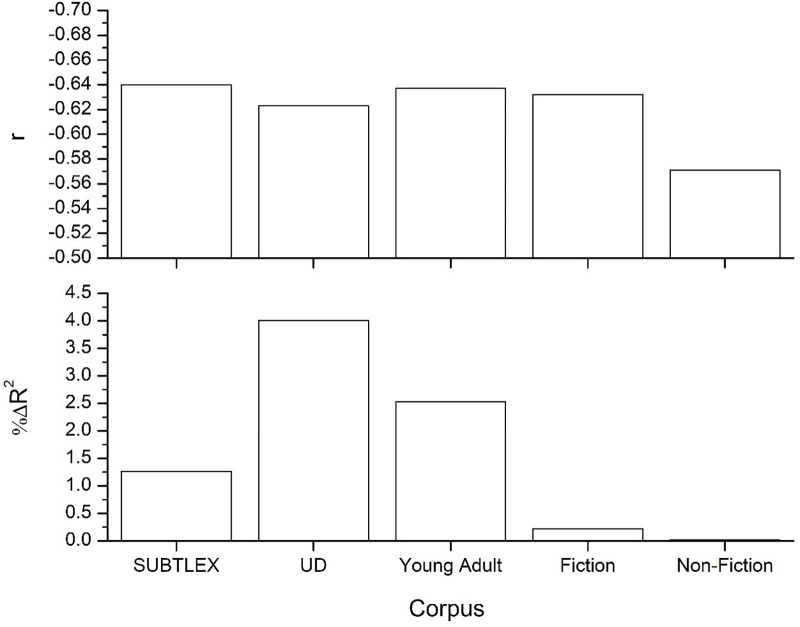
Top panel displays the correlation between word frequencies from the various corpora to lexical decision data from the English lexicon project (Balota et al., 2007), while the bottom panel displays the amount of unique variance each word frequency count accounts for. All correlations significant at the *p* < 0.001 level, while the *R*^2^-change values were all significant at *p* < 0.001 except for the frequency values from the non-fiction corpus.

As a first demonstration that the language contained in the mined UD data is unique compared to other corpora, word frequency of the words contained in the English Lexicon Project (ELP; Balota et al., 2007) was collected from the UD definitions and examples. These frequency values were contrasted to the word frequencies from the standard SUBTLEX corpus (Brysbaert and New, 2009), and to word frequency values calculated from a collection of young adult, fiction, and non-fiction books, each of which contained at least 80 million or more words (see Johns et al., 2018a for more details on these corpora). The top panel of Figure 1 displays the correlation of these frequency values to the z-transformed lexical decision reaction time data from the ELP. This figure shows that all word frequency values, with the exception of the non-fiction corpus, provide relatively equal fits to the lexical decision data.

Of course, there is a great deal of shared variance between these frequency values. In order to measure the predictive power of the different frequency values, a linear regression was used to quantify the amount of unique variance accounted for by word frequency counts from the different corpora. The analysis is standard and provides a measure of the predictive gain (i.e., measured as percent Δ*R*^2^ improvement) for one predictor over another competing predictor (see Adelman et al., 2006; Johns et al., 2012a, 2016b; Jones et al., 2012). The results of this analysis are contained in the bottom panel of Figure 1, and was done over words that were contained across all five corpora (*n* = 33,143). This figure shows that the frequency values from the UD actually account for the greatest amount of unique variance across the five corpora, followed by young adult novels and the SUBTLEX frequency values. The UD frequency values accounts for the most variance likely because it contains a considerably different type of language as compared to the other corpora (i.e., the other corpora have greater levels of redundancy in terms of the language that they contain and so cancel each other out to a great degree). This simulation demonstrates that the lexical data contained in the UD is of high quality and does offer good, and unique, fits to lexical behavior. These frequency values are publically available^2^.

**FIGURE 2:**
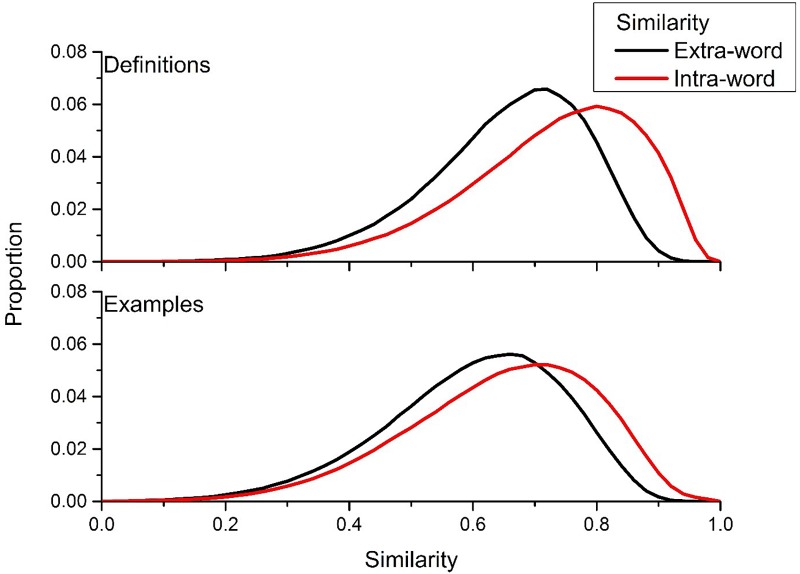
Intra- and extra-word similarity distributions for UD definitions **(top)** and example usages **(bottom)**. The positive shift of the intra-word similarity distribution in comparison to the extra-word similarity distribution signals that there is internal consistency in the meaning of the definitions and example usages that UD users are generating.

However, the main goal of this article is to assess the variability in meaning in the definitions that were generated to different cue words. Figure 2 contains the intra- and extra-word similarity distributions for both the definition (top panel) and example usages (bottom panel) derived from the UD data. The intra-word similarity distribution for the definitions contained approximately 2.6 million similarity comparisons, while there were approximately 2.4 million comparisons for the intra-word distribution for the example usages. Figure 2 shows that the definitions and examples that the UD users constructed do have internal consistency: the intra-word similarity distribution is positively shifted compared to the extra-word similarity distribution. The mean difference between similarity values from the intra-word and extra-word distribution was greater for definitions (0.071) than example usages (0.045), suggesting that attempting to define a word’s meaning allows for a person to produce a more discriminative utterance.

In standard experimental psychology, significance testing (e.g., ANOVA) is used to ensure that there is a significant difference between the means of two conditions (e.g., intra-word vs. extra-word similarity in this case). However, when using such large sample sizes as was done here, significance testing becomes trivial. For example, using a univariate ANOVA, the difference between the two conditions contained in the top panel of Figure 2, is significant with an *F*(1,3,626,457) = 147,471.2, *p* < 0.001. Even when this distribution is dropped to have 10,000 randomly selected comparisons from each condition, there is still an extremely significant difference of *F*(1,19,999) = 1,004.58, *p* < 0.001. Indeed, in this last comparison *p* = 3.72 × 10^-215^. The reason why significance testing becomes trivial when dealing with large sample sizes is because statistical techniques like ANOVA were designed to estimate the shape (e.g., a normal distribution from a sample mean and standard deviation) of a population’s behavior when sampling from that population, and significance is then determined by the distance between two constructed normal distributions (in the case of a two-factorial experiment). When extremely large sample sizes are used, the assumption that one is sampling becomes invalid, and instead it is possible to directly visualize the population under question (in this case, the semantic similarity of definitions and example usages produced to words in the UD). Due to this, any noticeable difference seen in the following simulations can be assumed to be a highly significant difference across conditions.

One additional question about the distributions contained in Figure 2 concerns the overlap between the intra-word and extra-word similarity distributions. The roughly Gaussian shape of these distributions comes from the assumptions of the BEAGLE model (see Jones and Mewhort, 2007). The underlying lying representation of BEAGLE comes from the summation of Gaussian vectors. This results in similarity distributions that have more spread and normality than other representation assumptions (see Johns and Jones, 2010 for direct examinations of this issue). It is possible that using a different distributional semantic model could result in more discriminated distributions. However, given the significant differences described in the above described paragraph, it is likely that BEAGLE is giving a good accounting of the differences between the intra- and extra-word similarity distributions.

This initial analysis suggests that even though many of the word and phrases that are being defined in the UD are relatively rare and not of common parlance, people do have a common understanding of the meaning of those words, at least relative to random similarity across both UD definitions and examples.

However, the comparison contained in Figure 2 only shows that the definitions and examples that UD users are generating offers unique information about the meaning of that word, not necessarily how informative each definition is about a word’s meaning. The standard position of distributional modeling is that each episodic experience that a person has with a word offers diagnostic information about the meaning of that word (Landauer and Dumais, 1997; McDonald and Shillcock, 2001; Jones, 2018). Across episodic experiences with language, this allows for a distributional model to form meanings to words that are discriminable from other words.

In order to assess how informative a specific lexical experience is to forming a semantic representation, it is necessary to compare the UD data to more standard types of language. To accomplish this, intra-word and extra-word similarity distributions were derived from corpora of fiction and non-fiction books. This analysis will contrast how unique a sentence from a standard corpus is to forming a word’s meaning, compared to the UD definitions and examples. Each corpus had 10 million sentences and these sets of fiction and non-fiction books did not occur in the training corpus (attained from Johns et al., 2018a). Given that many of the cue words from the UD data likely would not appear in these corpora, a new set of comparison cue words was constructed by taking the words that were contained in the both the English lexicon project (Balota et al., 2007) and were also cue words that had definitions and examples generated in the UD data. This resulted in a new comparison word set of 11,083 words, which had 71,850 definitions and 45,897 examples contained in the UD data.

To attain the required comparison sentences from the fiction and non-fiction corpora, all sentences that each cue word occurred in was recorded. Like in the analysis of the UD data, the maximum number of sentences a single word could have was 150. Representations of these sentences were constructed with equation (4), equivalent to how UD definitions/examples were analyzed in Figure 2. In order to make the comparison more equal, both the UD definitions/examples and sentences from the two corpora were capped at having a maximum of ten content words and a minimum of five content words. Additionally, the actual word being analyzed was removed from definitions/examples or sentences (equivalent to what was done previously for definitions/examples in the UD data), so that it is not simply the presence of the cue word in the sentences that is causing a shift in the similarity distributions. Intra- and extra-word similarity distributions were then computed for the UD data and the fiction and non-fiction sentences. For the UD definitions there was approximately 675,000 comparisons in the intra-word similarity distribution, while for the UD examples there was approximately 330,000 comparisons. For the fiction sentences there was approximately 1.73 million comparisons in the intra-word similarity distributions, while for the non-fiction corpus there were 1.34 million comparisons.

**FIGURE 3:**
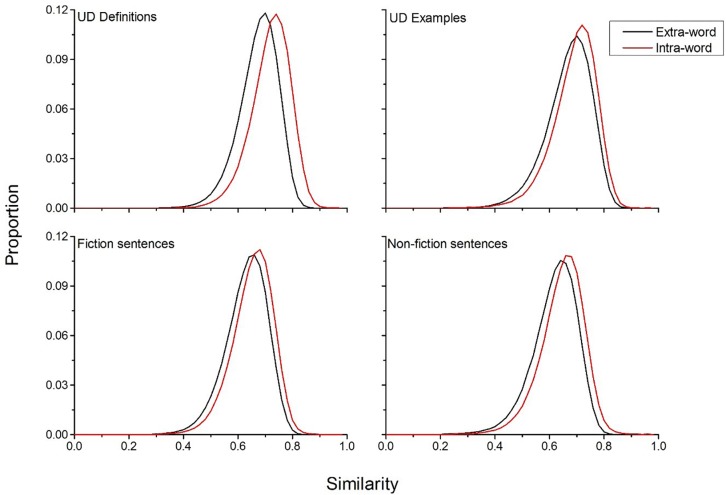
Comparison of extra- and intra-word similarity for the UD definitions and examples and sentences from the fiction and non-fiction corpora. The results of this simulation show that the definitions and example usages that UD users are at least as discriminative for a word’s meaning as sentences from standard natural language corpora.

Figure 3 displays the results of this simulation. This figure shows that for all four comparisons, the intra-word similarity distribution is shifted positively compared to the extra-word distribution, demonstrating that the sentences that a word occurs in offers unique information about the meaning of a word for both the UD data and the fiction and non-fiction corpora, as would be expected. However, the UD definitions provide as much or more discriminatory information than the fiction and non-fiction corpora, with a mean difference of 0.045 between the two distributions, while the UD examples had a mean difference of 0.022. The fiction sentences had a mean difference of 0.026, and the non-fiction sentences had a mean difference of 0.029. This suggests that when UD users engage in a process where they are attempting to communicate the meaning of a word to other people, they are able to generate language that is considerably informative about the meaning of that word. Indeed, the UD definitions contain more discriminative information than standard text types (this does not necessarily entail that the definitions are good, but instead that they are unique for that word).

**FIGURE 4:**
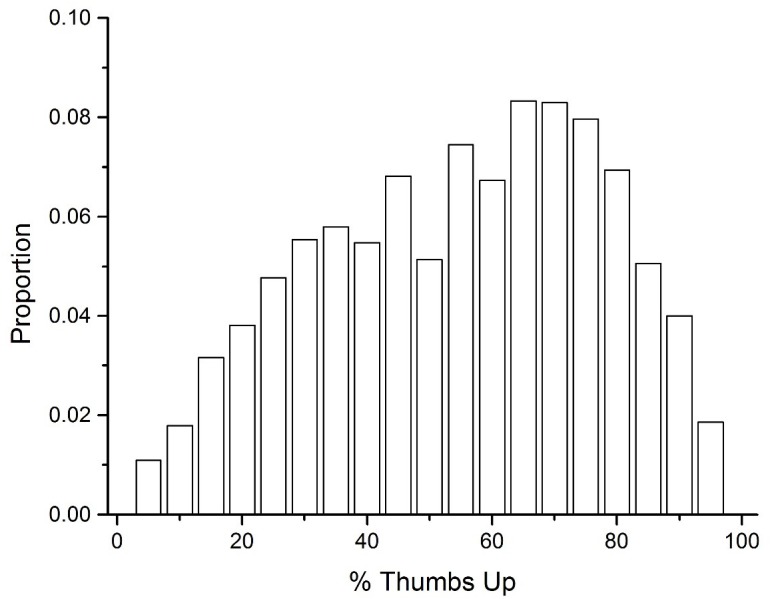
A histogram of the percent thumbs up for all UD definitions.

As described previously, another source of UD data that was collected was the number of thumbs up and thumbs down that other users gave to UD definitions. The first step to analyzing this data was to transform these ratings into percent thumbs up. A percentage value less than 50% would signal that there were more thumbs downs given to a definition than thumbs up. The mean percent thumbs up was 52.2%, signaling that there is a slight bias toward rating definitions positively. Figure 4 displays a histogram of this data across all of the definitions collected. This figure demonstrates that there is considerable variability in how accurate UD users believe definitions generated to cue words are, with the distribution being slightly negatively skewed.

**FIGURE 5:**
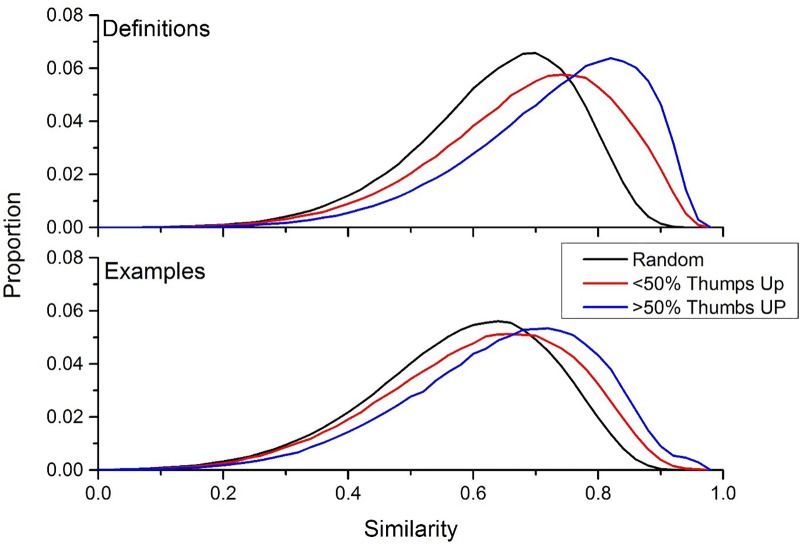
The intra-similarity distributions for definitions that have a percent thumbs up less than 50% (red line) and percent thumbs up greater than 50% (blue line). This result indicates that definitions that receive positive reception by the UD community have more internal consistency than definitions that are negatively received. This same trend holds for example usages, but the effect is not as large.

To determine whether there is a connection between the definitions that users think are accurate (i.e., definitions that have a greater percentage of thumbs up ratings), the intra-word similarity distributions were recalculated by taking the similarity between definitions/examples for a word that had a percentage thumbs up rating of less than 50%, and the intra-word similarity for definitions/examples that had a percentage thumbs up rating greater than 50%. Figure 5 contains the results of this simulation, for definitions (top panel) and examples (bottom panel). Additionally, the extra-word similarity distribution from Figure 2 were included in this figure to provide a comparison distribution. This figure shows a large effect of user preference on word meanings: positively rated definitions (i.e., definitions that had more thumbs up than thumbs down) were much more similar to each other than definitions that had negatively rated definitions. The same trend was found for example usages, but the effect was not as large.

An alternative definition of this finding is that the representations that BEAGLE is forming about the definitions is capable of discriminating well-constructed definitions (i.e., definitions that had a greater amount of thumbs up ratings) from poorly constructed definitions (i.e., definitions that had a greater amount of thumbs down ratings).

The results contained in Figure 5 suggests that the UD community at large has an idea of what a word means, as the definitions that are more likely to be accepted by the community have a greater level of consistency than the definitions that are considered poor. To get a better understanding of this trend, the mean similarity rating was calculated for the following percent thumbs up bins: 0–20, 20–40, 40–60, 60–80, and 80–100%. Although this splits the size of the intra-word similarity distributions considerably, there were still at least 10,000 comparisons at each comparison level. Additionally, in order to determine how this effect changes by word class, the mean similarity ratings of cue words from the ELP (used in Figure 3) was also calculated. These words were removed from the other UD cues.

**FIGURE 6:**
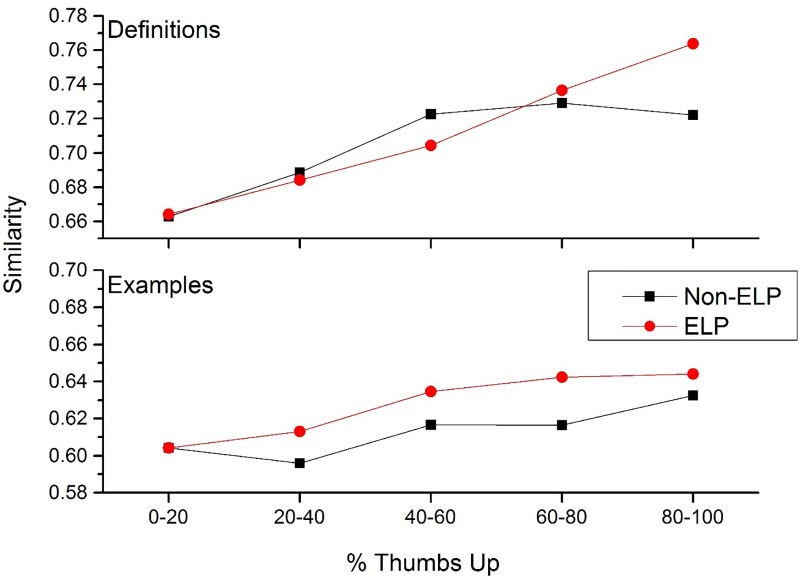
The increase in definition and example usage similarity as an effect of increasing percent thumbs up for cue words from the English lexicon project (red lines) and cue words that were not in the English lexicon project (black lines).

The results of this simulation is contained in Figure 6, for both definitions (top panel) and examples (bottom panel). For the definitions, there is a clear divergence of the ELP from the UD cues. For the ELP words, there is a constant increase in similarity among the definitions as percent thumbs up increases. This again shows that UD users have a clear preference for certain word meanings: the most positively rated definitions are also the ones that are the most similar. This makes sense for words from the ELP, as users likely have had a great deal of experience with these words, and thus have accurate expectations about what those words mean.

However, this trend does not hold for UD cue words. Definitions that have a greater percentage of thumbs down votes have lower level of intra-similarity, but this trend does not continue for definitions that have a greater percentage of thumbs up votes. This suggests that there is considerable ambiguity for what users consider to be the best definition of a word when those words are not well known. That is, there are a number of possible definitions that users are willing to accept for UD words, unlike the more common words contained in the ELP (e.g., users likely have a stronger preference for a definition to the word *dance* compared to the word *jocking*).

For the example usages generated, there was a different trend. There was a small increase in the intra-similarity of examples for both ELP and UD cue words, as percent thumbs up increases. This suggests that as users produce definitions that are more acceptable to the UD community, the more similar their example usages become. Thus, even though the definitions to UD cue words may not have the same consistency as definitions from the ELP, how they actually use those words in a generated example are relatively more similar to each other, compared with examples generated by users had more negatively received definitions.

The results of Figure 6 suggest that words that are well-known have more internal consistency in the definitions that are produced for them. A recent mega-dataset collected by Brysbaert et al. (2018) on word prevalence provides an ability to test this hypothesis directly. The word prevalence data of Brysbaert et al. (2018) was collected using a crowd-sourcing methodology, and was collected across hundreds of thousands subjects. The subjects were asked simply whether they knew that a word was a word. The main data type collected is the probability that the subjects knew a word was a word or not. Brysbaert et al. (2018) found that there is considerable variability in knowing whether a string is a real word – for example, 99% of people know that *bleak* is a word, but only 21% of people recognize that *aardwolf* is a real word. To test whether less prevalent words lead to more variable definitions, the words from Brysbaert et al. (2018) were split into upper and lower quartile according to probability known. The lower quartile consisted of words that were known by 0–67% of subjects (*n* = 15,150), and the upper quartile consisted of words known by 98–100% of the population (*n* = 16,875). For the words in the lower quartile, there were 1,128 cues contained in the UD data with 10,014 definitions, while for words in upper quartile there 7,126 cues contained in the UD data with 65,794 definitions. This led to 10,014 similarity contrasts for the lower quartile words, and 853,549 similarity contrasts for the upper quartile words. For example usages, there were 902 cues from the lower quartile words, with 8,905 different examples and 36,461 similarity contrasts. For the upper quartile words, there were 6,551 cues, with 56,128 different examples, and 665,324 contrasts.

**FIGURE 7:**
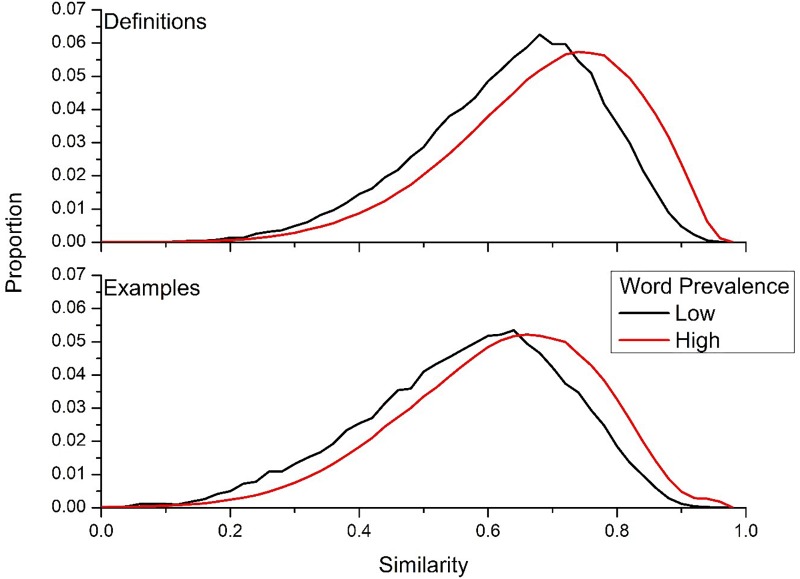
Similarity distributions for words that ranked low on the word prevalence measure attained from Brysbaert et al. (2018), and words that that had a high prevalence measure.

Figure 7 contains the histogram of the intra-word similarity distributions of the lower and upper quartile words for both definitions (top panel) and example usages (bottom panel). This figure confirms the finding of greater internal semantic consistency for words that are more well-known by the general population, as the definitions and example usages from the upper quartile of probability known are considerably more consistent than words from the lower quartile. The mean difference for definitions was 0.048, while it was 0.051 for examples. This suggests that words that are known by the general population have more consistent internal semantic representations across language users, resulting in more similar definitions and example usages from UD users.

## Discussion

The overarching goal of this article was to examine the consistency and preference that people have for word meanings. This was accomplished by mining a large amount of data from a crowdsourced dictionary, namely the Urban Dictionary. The data included definitions and examples to hundreds of thousands of different cue words. To assess the similarity of the definitions and examples that users of this dictionary generated to different cue words and phrases, a standard distributional model was used. It was found that there was considerable internal semantic consistency to the definitions and examples that UD users generated, even though many of the words being defined would likely not be known by the general population of language users. Indeed, it was found that the definitions users generated were more distinctive for a word’s meaning than writings from more standard language types.

An additional source of data that was collected from the UD was the ratings (as number of thumbs up and thumbs down) that other UD users gave to a specific definition. It was found that the definitions that receive the greatest percentage of positive ratings had more internal consistency compared to definitions that had a greater percentage of negative ratings. This suggests that the UD community at large has expectations about what a cue word means, resulting in positively rated definitions being more similar to each other. Furthermore, it was found that for standard words of English (defined as being words contained in the English lexicon project), there was a stark increase in the similarity of definitions as the percent of positive ratings increased. This suggests that for words that are well known or familiar, people have correspondingly strong preferences for those words. However, this trend did not hold for other cue words from the UD: at positive levels of positive ratings for these words there was no trend of increasing intra-similarity of definitions, meaning that the UD community was equally receptive to multiple alternative definitions for the same word.

The finding of word preferences in the UD data is consistent with the perspective that language is a complex adaptive system, a general perspective on language processing and the cultural evolution of language (e.g., Kirby et al., 2007; Christiansen and Chater, 2008; Beckner et al., 2009; Tomasello, 2009; see Johns and Jones, 2015; Jamieson et al., 2018 for computational models of semantics and language processing that embody this perspective). Adaptive theories of language propose that the acquisition and use of language is based in the past interactions that people have had with others in their social environment. For example, in the development literature it has been shown that the majority of the utterances that children produce is directly related to the utterances that they have heard (e.g., Lieven et al., 1997). The findings of this article support this perspective, as it is not the case that any definition contained in the UD was accepted by the UD community, instead only those that cohere to other language user’s expectations, conceptions, and beliefs are given a positive reception. That is, if a definition does not cohere with the past experience that a person has had with a word, then it is given a negative reception (in the case of UD data, this means giving a definition a thumbs down). The ability for people to reject word meanings sets constraint on adaptive theories of language, as presumably those definitions that are the most well received by the community are more likely to be propagated by members of that community using that acquired meaning. The end result of this propagation sets the stage for distributional semantics, where there are overlapping patterns in the usage of words across the users of a language that distributional learning mechanisms can exploit (see Johns and Jones, 2015 for a similar result using artificial agents). As is shown in Figure 5, definitions that are given a more positive reception by the UD community tend to be more similar to each other, likely signaling the beginning stages of meaning formation for those words.

The current work is a prime example of both the promise and limitations of big data approaches to psychology. On a positive note, this research shows the utility that distributional models of semantics have as general tools in the analysis of lexical behavior. Typically, these models are used to understand the connection between lexical experience and knowledge acquisition (Landauer and Dumais, 1997), with different models exploiting alternative methods at learning this information (e.g., Griffiths et al., 2007; Jones and Mewhort, 2007). That is, they have been mainly used as theories to better understand semantic memory. The results of this article point to an alternative use of these models, namely to quantify linguistic information in order to gain an understanding of human behavior across diverse tasks (e.g., Johns et al., 2012b, 2018b; Taler et al., 2013, 2019; Johns and Jamieson, 2018). Distributional models provide an ability to quantify large amounts of data, which can then provide insight into behavior and underlying cognitive processes.

However, the limitation of the approach used here is a lack of control, a hallmark of traditional work in the psychological sciences. Instead of control, the scale of the data allows for the natural noise in data to be understood by visualizing the distributions of the requisite comparisons under question, as was done in this article. Thus, it is possible to distinguish large scale trends in human behavior (e.g., the effect of user preference on definition similarity in Figure 5), but it is difficult to isolate the causes of this behavior, due to lack of control over the task. As discussed previously, this is due to the fact that big data in psychology is largely an abductive science (e.g., Haig, 2005; Johns et al., in press), where hypothesis are generated to found data. The goals of abduction align well with many trends in mega-studies of human behavior, with the first major study being the ELP (Balota et al., 2007) as discussed, followed by many other types of data, such as the semantic priming project (Hutchison et al., 2013), word prevalence (Brysbaert et al., 2018), reading times (Cortese et al., 2018), and embodied characteristics of words (Tillotson et al., 2008), to name just a few (see Johns et al., in press, for a review of some of these projects). The hope of these projects is that the patterns contained in these large sets of data allow researchers to generate better theories of the behaviors under question.

Specifically, there are a number of questions about lexical semantics that this article raises. One is the connection between word frequency and word meanings. As Figure 7 shows, words that are more well-known (and hence, likely experienced more) have more consistent definitions generated for them within the UD. This begs the question as to whether a similar pattern exists across the lexicons of language users, where people have more consistent patterns of word meanings for highly experienced words, but relatively different representations for words that are experienced fewer times. This question also has general implications for how distributional models of semantics are trained and tested, since if there is a greater variability in meaning for low frequency words, then the specific training corpus is going to have a large impact on the performance of the model on those word types, compared to more frequent words.

A related question concerns the graded nature of word meanings. As Figure 5 shows, for definitions that are positively received (meaning that they received more thumbs up rating than thumbs down), these words have a greater level of consistency compared to definitions that were not as well received. However, there is still a great deal of variability in these definitions, suggesting that there is a range of definitions that people find acceptable for word meanings. This suggests that word meanings may be more dynamic than previously thought, a challenge for distributional models of semantics.

The findings of word meaning consistency and preference in this article are prime examples of abductive work. In response to a large dataset of human behaviors (and one that is considerably noisier than controlled datasets like the ELP), a set of behavioral observations were abducted. Namely, it was found that even at the beginning of meaning formation, a community of language users have consistency in their conception of what a word or phrase means. Additionally, it was found that language users have strong preferences for word meanings. For big data analytics to be a productive methodology within psychological science, these observations need to be used to inform controlled and targeted empirical research, in order to get a better an understanding of the mechanisms that underlie semantic cognition.

## Author Contributions

The author confirms being the sole contributor of this work and has approved it for publication.

## Conflict of Interest Statement

The author declares that the research was conducted in the absence of any commercial or financial relationships that could be construed as a potential conflict of interest.

## Footnotes

1 Specifically, the command http://api.urbandictionary.com/v0/random was used to retrieve 10 random definitions at once. Definitions were then collected and collated over a period of 2 months.

2 The word frequency values from the UD corpus are contained as Supplementary Material to this article. This file contains the frequency values from the combined word set from the ELP and the word prevalence measure of Brysbaert et al. (2018).

## Supplementary Material

TABLE S1 The first row of the table contains the combined words from the Balota et al. (2007) and Brysbaert et al. (2018) studies, while the second row contains the log frequency from the UD corpus.
